# Plasma concentrations of IL-8, IFN-γ and IL-1β in schizophrenia patients with subgroup analysis of first episode drug naïve patients

**DOI:** 10.1192/j.eurpsy.2024.581

**Published:** 2024-08-27

**Authors:** M. Markovic, M. Stasevic, M. Stojkovic, M. Velimirović Bogosavljević, T. Stojkovic, M. Zivkovic, I. Stasevic Karlicic, T. Nikolic, S. Totic Poznanovic, N. Petronijevic

**Affiliations:** ^1^Clinic for Mental Disorders “Dr Laza Lazarević”, Belgrade, Serbia, Belgrade; ^2^Clinic for neurology, University Clinical Centre of Nis, Nis; ^3^Institute for medical and clinical biochemistry; ^4^Faculty of Medicine, University of Belgrade, Belgrade; ^5^Faculty of Medicine, University of Priština – Kosovska Mitrovica, Kosovska Mitrovica; ^6^Clinic for Psychiatry, University Clinical Centre of Serbia, Belgrade, Serbia

## Abstract

**Introduction:**

Increased plasma concentrations of proinflammatory cytokines are found in chronic schizophrenia patients, patients with first episode and in individuals with high risk for psychosis. The most replicated findings are increased concentrations of IL-6, TNF-α and IL-1β through different phases of the disorder while the results for two important proinflammatory cytokines IL8 and IFN-γ were not consistent.

**Objectives:**

Primary objective of this study was to assess differences in concentrations of IL-8, IFN-γ and IL-1β between schizophrenia patients and healthy controls, Secondary objective was to explore differences in first episode drug naïve patients.

**Methods:**

We measured plasma concentrations of IL-8, IFN-γ and IL-1β in 64 healthy controls and 64 schizophrenia patients during acute exacerbation and remission phase. 25% were drug naive first episode schizophrenia patients. The patients were matched by age, sex and body mass index and exclusion criteria included obesity class 2 or higher, any concomitant organic mental or neurological disorder, acute or chronic inflammatory disease, and use of immunomodulatory drugs or psychoactive substances.

**Results:**

Levels of IL-8 were significantly lower in patients with schizophrenia in acute phase and remission compared to healthy controls (p=0,009 for acute phase and p=0,020 for remission). There was no significant difference in the levels of INF-γ and IL-β between schizophrenia in acute phase and remission and healthy controls (p>0,05). In schizophrenia patients there was no difference in the levels of INF-γ, IL-β and IL-8 between acute phase, remission and healthy controls (p>0,05). There was no difference in plasma levels of IL-8, IFN-γ and IL-1β between first episode drug naïve and previously treated schizophrenia patients.

**Conclusions:**

Our research did not find disturbance of plasma levels of IFN-γ and IL-1β in schizophrenia patients, although the increase of IL-1β was the most replicated finding up to date. Interestingly and contrary to expected the finding of significantly decreased levels of IL-8 in schizophrenia patients requires further research since IL-8 plays a vital role in the inflammatory pathway and may be implicated in cognitive dysfunction.

**Disclosure of Interest:**

None Declared

